# The Role of Endocrine System in the Inflammatory Process

**DOI:** 10.1155/2016/6081752

**Published:** 2016-09-29

**Authors:** Christian Bowman-Colin, Luis A. Salazar, Joilson O. Martins

**Affiliations:** ^1^Dana-Farber Cancer Institute and Harvard Medical School, Boston, MA, USA; ^2^Center of Molecular Biology and Pharmacogenetics, Faculty of Medicine, Universidad de La Frontera, Temuco, Chile; ^3^Department of Clinical and Toxicological Analyses, Faculty of Pharmaceutical Sciences, University of São Paulo, São Paulo, SP, Brazil

Inflammation is a general tissue response to a wide variety of stimuli. In situations in which inflammation is not properly regulated, inflammatory response may be exaggerated or ineffective, leading to immune dysfunction, recurrent infections, and tissue damage, both locally and systemically. Various hormones, cytokines, vitamins, metabolites, and neurotransmitters are known to be key mediators of the immune and inflammatory responses in endocrine as well as in paracrine fashions. Therefore, exploring the mechanisms underlying the production and response to these mediators might broaden the horizons for the development of novel therapeutic options that target disease states in which the immune/inflammatory responses are compromised or dysregulated.

This special issue covers the most current research aimed at elucidating the cellular and molecular mechanisms underpinning the endocrine/paracrine networks of regulatory immune mediators and their targets.

In this journal edition in disease states, Y.-S. Lee and H.-S. Jun reviewed the current status of glucagon-like peptide-1- (GLP-1-) based therapies and their impact on the treatment and management of type 2 diabetes mellitus. GLP-1 is an incretin hormone mainly secreted by intestinal L cells in response to nutrient ingestion, which has beneficial effects for glucose homeostasis by stimulating insulin secretion from pancreatic beta-cells, delaying gastric emptying, decreasing plasma glucagon, reducing food intake, and stimulating glucose catabolism. Beyond their metabolic effects, it is reviewed herein that GLP-1-based therapies have displayed anti-inflammatory properties through promoting downregulation of proinflammatory responses in a cell-autonomous as well as a systemic manner, especially in the context of inflammation-related diseases.

A. Mancini et al. report in this issue that thyroid hormones play particularly important roles in the antioxidant balance, since both hyper- and hypothyroidism have been shown to be associated with oxidative stress (OS) in humans and animals. In this context, the pathophysiological mechanisms of the nonthyroidal illness syndrome (NTIS) typically manifest as reduced conversion of thyroxine (T4) to triiodothyronine (T3) in several acute and chronic systemic conditions. This syndrome, along with the deiodinases that catalyze the conversion of T4 to T3, is reviewed herein.

Female development and reproductive function is well documented to be modulated by estrogens. In particular, 17*β*-estradiol (E2) is the main sex hormone regulating reproduction in females. However, E2 is also deeply involved in several other pathologies, such as cancer and autoimmune and infectious diseases, in which the innate immune response is a key player. I. Medina-Estrada et al. reported in this issue that E2 induces anti-inflammatory responses of bovine mammary epithelial cells during* S. aureus* internalization and that effect is dependent, at least in part, on the estrogen receptor *α* (ESR*α*).

Like estradiol, progesterone levels fluctuate dramatically during pregnancy. In this issue, M. Wu et al. report that the known increase in serum progesterone levels during pregnancy exacerbates gingival inflammation. This effect of progesterone is shown to be independent of crevicular fluid levels of both interleukin-1*β* (IL-1*β*) and tumor necrosis factor-*α* (TNF-*α*).

Diabetic retinopathy (DR) is the most common microvascular complication of diabetes and is a leading cause of blindness across the globe. Genetic predisposition has been found to contribute to DR pathology, since specific haplotypes cosegregate with disease onset within affected families. M. M. Yang et al. reported herein that a polymorphism in the C5 gene (rs17611) represents a novel putative susceptibility locus for DR, particularly predisposing to the clinically relevant proliferative DR subtype. On the other hand, it was shown that polymorphism of SERPING1, which encodes for one well-known component of the Complement system, has only marginal to no contribution to the development of DR.

Endoplasmic reticulum (ER) stress facilitates fibrotic remodeling through the promotion of inflammatory responses. Aldosterone (Aldo), a known ER stressor, is thought to be involved in fibrotic renal injury by upregulating the production of inflammatory mediators such as IL-1*β* and IL-6. H. Guo et al. reported an important role for Aldo responses in ER stress and renal inflammation in the pathogenesis of renal fibrosis. In addition, the ER stress can be inhibited by Tauroursodeoxycholic Acid (TUDCA) and this effect is associated with downregulation of collagen I, collagen IV, fibronectin, transforming growth factor-*β* (TGF-*β*) expression, and Nlrp3 inflammasome markers such as the apoptotic speck protein (ASC), IL-1*β*, and IL-18. Altogether, these findings suggest that these inflammatory pathways are involved in Aldo-induced chronic kidney disease.

In summary, the original research articles and literature reviews featured in this special issue will hopefully enhance our knowledge about the roles of the endocrine system in the inflammatory process, shedding light on potential avenues for the development of novel therapies.

